# Islet cilia and glucose homeostasis

**DOI:** 10.3389/fcell.2022.1082193

**Published:** 2022-12-01

**Authors:** Isabella Melena, Jing W. Hughes

**Affiliations:** Division of Endocrinology, Metabolism and Lipid Research, Department of Medicine, Washington University School of Medicine, Saint Louis, MO, United States

**Keywords:** primary cilia, beta cells, pancreatic islets, paracrine signaling, glucose regulation

## Abstract

Diabetes is a growing pandemic affecting over ten percent of the U.S. population. Individuals with all types of diabetes exhibit glucose dysregulation due to altered function and coordination of pancreatic islets. Within the critical intercellular space in pancreatic islets, the primary cilium emerges as an important physical structure mediating cell-cell crosstalk and signal transduction. Many events leading to hormone secretion, including GPCR and second-messenger signaling, are spatiotemporally regulated at the level of the cilium. In this review, we summarize current knowledge of cilia action in islet hormone regulation and glucose homeostasis, focusing on newly implicated ciliary pathways that regulate insulin exocytosis and intercellular communication. We present evidence of key signaling proteins on islet cilia and discuss ways in which cilia might functionally connect islet endocrine cells with the non-endocrine compartments. These discussions aim to stimulate conversations regarding the extent of cilia-controlled glucose homeostasis in health and in metabolic diseases.

## Introduction

**FIGURE 1:**
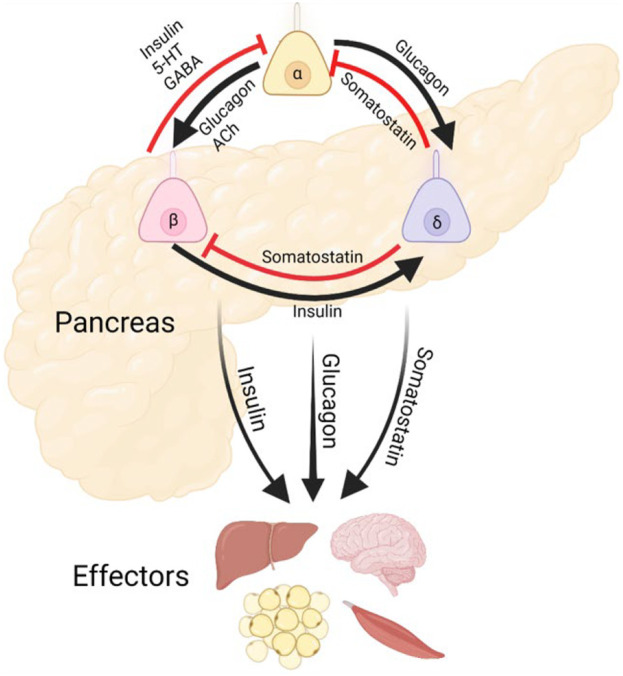
Pancreatic Islet Cilia Regulate Glucose Homeostasis. Schematic depicting putative roles of primary cilia in coordinating islet hormone secretion and action, as well as potential output pathways affecting other metabolic organs. Cilia are expressed in each of these islet cell types and may regulate both cell-intrinsic functions and intercellular crosstalk. Alpha and delta cell cilia are less studied and do not yet have established roles in paracrine regulation.

Blood glucose in the human body is normally maintained in a tight range of 4.4–7.0 mM (80–126 mg/dl), thanks to the precisely coordinated secretion of insulin from β-cells, glucagon from α-cells, somatostatin from δ-cells, and their combined action on target tissues. Pancreatic islet hormones have both long- and short-range actions as endocrine or paracrine factors, respectively. When released into the blood stream, insulin and glucagon act on target tissues including muscle, adipose, and liver to stimulate glucose uptake or gluconeogenesis. Locally within the islets, these hormones along with somatostatin act as paracrine cues to influence neighboring cell behavior (Figure 1). A classically defined β-cell disease, diabetes is increasingly seen as a collective dysregulation of all islet endocrine cells, as well as disrupted communication between the pancreas and other metabolic organs (Bouzakri et al., 2011; Dunmore and Brown, 2013; Watt et al., 2019; López-Bermudo et al., 2022).

The release of glucoregulatory hormones is modulated by many extracellular signals, some of which are organized and transduced by the primary cilium, a cell-surface organelle with sensory and signaling capacity. Primary cilia are expressed by the majority of islet endocrine cells, where they mediate not only hormone secretion but also intercellular communication and likely influence cellular decisions such as proliferation, differentiation, and regeneration. Loss of primary cilia structure or function, as seen in human ciliopathies like Bardet-Biedl and Alström syndromes, are associated with early-onset endocrinopathies including obesity and type 2 diabetes, clinically characterized by glucose intolerance and insulin resistance (Zaghloul and Katsanis, 2009; Hildebrandt et al., 2011; Hearn, 2019). A number of experimental mouse models have probed the mechanistic link between primary cilia and glucose control. Targeted deletion of primary cilia in pancreatic islets and particularly in β-cells disrupt insulin secretion and cellular crosstalk, producing systemic glucose dysregulation (Gerdes et al., 2014; Volta et al., 2019; Hughes et al., 2020; Cho and Hughes, 2022), while a growing list of ciliary genes are linked to diabetes and obesity in both humans and experimental models (Davenport et al., 2007; Berbari et al., 2008; Mukhopadhyay and Jackson, 2013; Kluth et al., 2019; Lodh, 2019; Walker et al., 2021). These data support a pathogenic link between ciliary defects and diabetes and underscore the need to better understand the mechanisms of cilia control of islet function. Here, we summarize what is known about pancreatic islet cilia in the context of glucose regulation and diabetes.

### Cilia-related metabolic disease

Primary cilia are dynamic sensors of nutrient abundance and are themselves regulated during the cell cycle and times of energy starvation (Boehlke et al., 2010; Pampliega et al., 2013). In muscle and adipose tissue, primary cilia are required for normal differentiation and tissue expansion and regeneration (Fu et al., 2014; Jaafar Marican et al., 2016; Hilgendorf et al., 2019). In neuronal tissues, primary cilia regulate feeding behavior and energy balance (Engle et al., 2021; Yang et al., 2021). Genetic mutations that disrupt ciliary structure or function in the whole body result in human ciliopathies with complex metabolic defects (Lee et al., 2015a; Lodh, 2019; Cho and Hughes, 2022). Two classic human ciliopathies, Bardet-Biedl (BBS) and Alström syndromes, manifest as youth-onset obesity, insulin resistance, and type 2 diabetes. While both affecting glucose metabolism, these diseases are mechanistically different and have distinct effects on beta cell function and mass disruption, as well as extra-pancreatic effects on insulin sensitivity and glucose utilization (Marion et al., 2012; Lodh et al., 2016; Han et al., 2018). Monogenic mouse models of both syndromes *via* multiple *Bbs* and Alms genes phenocopy aspects of human disease, manifesting with variable severities of glucose intolerance, abnormal fasting glucose, and elevated fed serum insulin (Collin et al., 2005; Eichers et al., 2006; Gerdes et al., 2014). These metabolic phenotypes have also been recapitulated by animal models of other key cilia genes (Zaghloul and Katsanis, 2009; Cho and Hughes, 2022). In particular, whole-body disruption of Ift88, an essential gene for ciliary assembly and maintenance, causes mice to lose tolerance of both short-term fasting and acute glucose challenge (Zhang et al., 2005), where the metabolic phenotypes seen in this *orpk* hypomorph mouse may also be related to its developmental exocrine pancreas deficiency. Whole-body deletion of Kif3a and Rfx3, regulators of ciliary transport and ciliogenesis, show similar metabolic disturbances including elevated fasting serum glucose as seen in Ift88 loss-of-function (Ait-Lounis et al., 2007; Davenport et al., 2007). While the Kif3a deletion produces increased fasting serum insulin (Davenport et al., 2007), Rfx3 deletion elicits a more pronounced defect in glucose-stimulated insulin secretion (GSIS) and glucose tolerance (Ait-Lounis et al., 2007).

As interpretation of early Ift88 models was limited by the lack of pancreas-specificity, subsequent targeted β-cell models were developed to address the issue. These include the conditional and constitutive mice that deleted Ift88 gene expression under the control of Pdx1-CreER and Ins1-Cre or Ins1-CreERT2 (βICKO and βCKO/βCKO-ERT2, respectively) (Volta et al., 2019; Hughes et al., 2020). These mice exhibited impaired GSIS and glucose intolerance without obesity, where the glycemic defects could be isolated to islet-intrinsic cilia effects rather than secondary effects from insulin resistance and hyperphagia-related obesity, where cilia plays a known role (Davenport et al., 2007; Volta and Gerdes, 2017; Yang et al., 2021). In the absence of primary cilia, cell-cell communication is disturbed in intact islets, as evidenced by altered juxtacrine ephrin signaling and decreased sensitivity to paracrine signals (Volta et al., 2019; Hughes et al., 2020). The latter manifests as loss of β-cell responsiveness to somatostatin, as well as dysregulated α-and δ-cell hormone secretion (Hughes et al., 2020). Human transcriptomic studies have identified cilia gene changes across islet cell types in type 2 diabetes, suggesting a link between islet cilia perturbations with human metabolic disease (Lawlor et al., 2017; Kluth et al., 2019; Walker et al., 2021). Taken together, the emerging data suggest that primary cilia play complex roles in islet cell glucose handling and cellular communication, both critical to whole-body metabolic homeostasis.

### Structural basis for cilia regulation of glucose metabolism

In both rodent and human islets, β-cells comprise the predominant cell type where both cell polarity and connectivity are crucial for coordinated insulin secretion. Morphological studies of intact islets show that β-cells establish direct physical connections with each other and with α- and δ-cells, typically as tubular arrays of cells that are seen as rosette-like clusters around the vasculature (Bonner-Weir, 1988; Weir and Bonner-Weir, 1990; Low et al., 2014; Bonner-Weir et al., 2015; Geron et al., 2015; Weitz et al., 2021). Within these clusters, cell polarity is defined by orientation to the vasculature – namely, the base abutting the arterioles and the apex pointing to a central draining vein, shared among cells of the rosette. The lateral sides of the β-cell, or so-called the edge domain, constitutes an important functional microdomain that mediates adhesion and signaling (Weir and Bonner-Weir, 1990; Geron et al., 2015). Primary cilia are preferentially located on this lateral surface of β-cells (Granot et al., 2009a; Geron et al., 2015; Gan et al., 2017), where they are seen as long, narrow projections among closely juxtaposed cell bodies (Cano et al., 2004; Collin et al., 2005; Zhang et al., 2005; Cano et al., 2006; Eichers et al., 2006; Ait-Lounis et al., 2007; Davenport et al., 2007; Granot et al., 2009a; Volta et al., 2019; Hughes et al., 2020). Cilia have a unique cytostructure comprised by a microtubule-based axoneme core surrounded by a ciliary membrane that is in communication with the plasma membrane. There is strict spatiotemporal control of protein trafficking to and from the cilium, and signaling pathways are organized in specific compartments within the axoneme, transition zone, and basal body (Fliegauf et al., 2007; Wheway et al., 2018). Classic ciliary signaling pathways including Hedgehog (Hh), Wingless/Int (Wnt), Notch, and transforming growth factor beta (TGF-β) all play essential roles in pancreatic development and mature pancreas function (Lodh et al., 2014; Fitzsimons et al., 2022; Koca et al., 2022; Nguyen et al., 2022; Szymanska et al., 2022; Xiao et al., 2022), and among these, Hh signaling is the most studied in pancreatic cell cilia (Zhang et al., 2005; Cervantes et al., 2010; Sanchez et al., 2022). Primary cilia also regulate β-cell size and polarity through ciliary proteins like LKB1 (liver kinase B1), a serine-threonine kinase and tumor suppressor that mediates cellular energy sensing and metabolism through AMPK (AMP-activated protein kinase) phosphorylation (Granot et al., 2009a; Boehlke et al., 2010).

**FIGURE 2:**
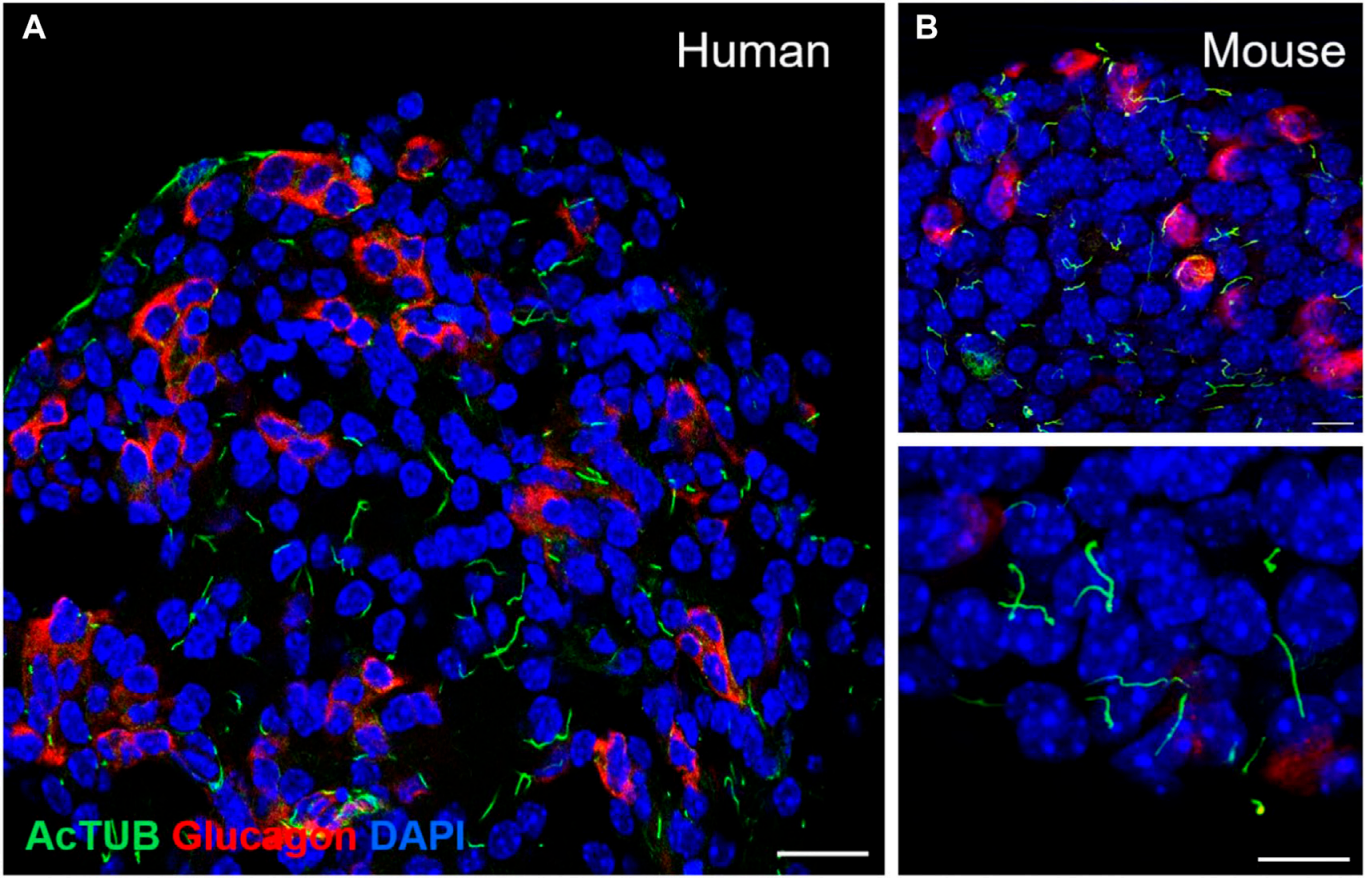
Detection of primary cilia in human and mouse islets by immunofluorescence staining. **(A)** Confocal image of a single-plane cross-section of a healthy human islet, showing primary cilia on both alpha cells (glucagon, red) and non-alpha cells (non-red). Acetylated alpha tubulin, cilia (green), nuclei (blue), scale 20 μm. **(B)** Primary cilia in wildtype B6 mouse islets: acetylated alpha tubulin (green), glucagon (red), nuclei (blue), single-plane images, scales 10 μm.

Most endocrine cells in the adult mouse and human islet express a single primary cilium, as do most cells in the mammalian body, and these are readily detectable by light microscopy (Figure 2). Notably, the ciliation cycle is coupled with the cell cycle, as cytokinesis typically requires cilia resorption, and the presence of primary cilia is thought to transduce cytostatic signals to the cell (Seeley and Nachury, 2010; Kim and Tsiokas, 2011). The lateral localization of the β-cell cilium also dictates its projection into the intra-islet canaliculi system, where cilia from adjacent cells can interact and form potential physical connections (Iwanaga et al., 2011; Gan et al., 2017). This luminal space contains interstitial fluid that connects the arterial to venous flow and represents a crucial glucose- and nutrient-rich source for the cells (Orci et al., 1989; Weir and Bonner-Weir, 1990; Iwanaga et al., 2011). Recent identification of dynein and associated motor proteins in islet primary cilia has challenged conventional classification of primary versus motile cilia and suggests that primary cilia may detect a greater sensory milieu given potential 3D mobility in this space (Cho et al., 2022; Sui, 2022).

Stable cilia-cilia interactions have been observed in cultured 3D kidney cells, lasting hours to days, occurring through glycoprotein-mediated membrane adhesion (Ott et al., 2012). We have observed similar encounters among human and mouse islet cilia (unpublished findings), while the significance of this behavior in islet physiology is yet unknown. We speculate that islet cell cilia mediate intercellular communication *via* potential physical swapping of biomaterials or by stabilizing membrane juxtapositions and thereby promoting formation of signaling complexes. In this setting, having cilia be *motile* would further promote cilia-cilia interactions among β-cells and non-β-cells alike, thus increasing the capacity for homotypic and heterotypic cellular coupling. Ciliary movement gives cells greater physical reach for signal detection, also allows multiple cilia to potentially orient themselves toward a shared sensory milieu, which could amplify regional responses and coordination to nutrients and paracrine factors. Lastly, signaling may be triggered by cilia movement itself, as physical deflection of the ciliary axoneme as a mechanosensor has been shown to evoke Ca^2+^ signals that affect downstream cell decisions in non-islet tissue (Nauli and Zhou, 2004; Nauli et al., 2008; Wann et al., 2012; Lee et al., 2015b; Delling et al., 2016), although these observations may be cell type- and experimental system-specific. These additional layers of cilia function need to be clarified in islets and may underlie individual and collective sensing by islet cells of their microenvironment.

### Ciliary protein trafficking and intercellular communication

The sorting and movement of proteins into and out of primary cilia is a tightly regulated process. Despite being continuous with the plasma membrane, a ciliary diffusion barrier at the basal body and transition zone maintains functional separation of the cilioplasm from the cytoplasm. Cargo vesicles dock and fuse at the ciliary base and their contents are transported by IFT complexes along the cilium (Hsiao et al., 2012; Madhivanan and Aguilar, 2014; Garcia et al., 2018). The BBSome, a functional complex of adapter proteins, serves to connect the IFT machinery to protein cargo including GPCRs, while *Bbs* mutations cause defects in ciliary protein entry and removal and lead to abnormal ciliary membrane composition (Nozaki et al., 2018; Wingfield et al., 2018). The recent demonstration of cilia-regulated GABA signaling, *via* ciliary localization of GABA receptor B1 but not B2 or GABA-A (Sanchez et al., 2022), drives home the point of specialized ciliary protein recruitment and signaling. Similarly, localization of SSTR3 but not the other SSTR isoforms to β-cell primary cilia, as well as dynamic recruitment of insulin receptor IR-A but not IR-B (Gerdes et al., 2014) speak to the fine-tuning of ciliary signaling that likely underlies autocrine and paracrine feedback loops *via* locally secreted islet hormones. There is reciprocal regulation between the cytoskeleton and primary cilia, where the actin network dynamically influences ciliogenesis, cilia trafficking, and ciliary length (Valente et al., 2010; Sharma et al., 2011; Phua et al., 2017; Copeland, 2020). The ciliary basal body itself is a microtubule organizing center whose assembly and dynamics directly regulate organelle function, cell division, migration, and polarity (Gong et al., 2004; Marshall, 2009; Luowei et al., 2021). There is limited experimental evidence for ciliary control of islet cell proteostasis or cytosolic protein trafficking, but we know from other tissue and cell types that cilia can initiate nutrient signaling and phosphorylation of downstream proteins *via* mTOR, AMPK, AKT, and are related to the autophagic flux (DiBella et al., 2009; Rosengren et al., 2018; Arora et al., 2020; Zhang et al., 2021). Additionally, cilia have been found to excrete bioactive extracellular vesicles, exosomes or ectosomes, which may serve as a means of longer range communication (Bloodgood et al., 1986; Dubreuil et al., 2007; Hogan et al., 2009; Wood et al., 2013; Wang et al., 2014; Wood and Rosenbaum, 2015; Long et al., 2016; Garcia et al., 2018). Electron micrograph studies have demonstrated ciliary ectosomes in gerbil islets (Boquist, 1970), while ciliary vesicle shedding in other species as well as any functional implications await elucidation.

Multiple physical means underlie primary cilia function as an interpreter of intercellular signals. As a membranous protrusion, primary cilia detect flow and bending, release and detect vesicles and other bioactive materials, and can form long-lasting contacts amongst themselves (Praetorius and Spring 2001; Ott et al., 2012; Delling et al., 2013; Wood et al., 2013; Lee et al., 2015b). In islets, given the tight physical proximity among cellular structures, primary cilia likely have the capacity to form direct contacts among neighboring α-, β-, and δ-cells in privileged domains where other cellular regions may lack the opportunity to interact (Gan et al., 2017; Lodh, 2019). If so, this could implicate primary cilia in the establishment and maintenance of functional and physical connectivity of islet α-, β-, and δ-cells, a mechanism that could serve to stabilize juxtacrine signaling or provide other means of contact-dependent sensing (Ott et al., 2012). The disruption of such processes may in part explain the aberrant hormone secretion and glucose response seen in cilia knockout islets (Volta et al., 2019; Hughes et al., 2020). Primary cilia are known to house a variety of adhesion molecules, including cadherins, integrins, and glycoproteins (Ott et al., 2012; Seeger-Nukpezah and Golemis, 2012). Stable direct contact *via* their cilia may allow cells to swap vesicles, ions, membranes, or other cellular contents that allow communication about the activity of neighboring cells. Islet α-, β-, and δ-cell primary cilia may each display different adhesion molecules, allowing their cilia to distinguish self- or non-self cell contacts. Correspondingly, individual islet cells may signal excitation or other changes in their cell state by altering ciliary movement or adhesive properties, allowing cells to selectively interact or distinguish “activated” cells from a dormant one. Given that we and others observe decreased islet cell mass in the absence of cilia (Cano et al., 2006; Ait-Lounis et al., 2007; Volta et al., 2019; Hughes et al., 2020), it is also possible that primary cilia mediate trophic signals among islet cells for the maintenance of α-, δ-, and β-cell mass and identity. Along these lines, there remain many interesting questions regarding the contact-dependent nature of cilia signaling and its physiologic purpose in islet cells.

### Ciliary G protein coupled receptors in islets

G protein-coupled receptors (GPCRs) encompass a major class of ciliary signaling pathways that promote cellular function and connectivity (Koemeter-Cox et al., 2014; Schou et al., 2015; Guo et al., 2017; Mykytyn and Askwith, 2017; Pala et al., 2017; Engle et al., 2021). In metabolic endocrine cells, primary cilia sense an array of extracellular stimuli *via* GPCRs localized to the ciliary membrane. Among these receptors are somatostatin receptor 3 (SSTR3), G-protein coupled receptor 161 (GPR161), 5-hydroxytryptamine (serotonin) receptor 6 (5HT_6_, HTR6), and melanocortin 4 receptor (MC4R) (Händel et al., 1999; Berbari et al., 2008; Mykytyn and Askwith, 2017; Kobayashi et al., 2020; Barbeito et al., 2021). In islets, the primary cilium represents a unique GPCR signaling domain, as multiple nutrient-sensing GPCR receptors have been discovered in islet α-cell and β-cell cilia (Wu et al., 2021). Two such receptors, free fatty acid receptor (FFAR4) and prostaglandin E receptor 4 (PTGER4), stimulate ciliary cAMP signaling and promote glucagon and insulin secretion. Inhibiting ciliary localization of these receptors *via* tubby-like protein 3 (TULP3), a ciliary trafficking protein, blocks glucagon or insulin secretion, suggesting that islet hormone secretion requires the coordination of these signaling pathways on primary cilia (Wu et al., 2021).

Other than nutrient cues, a number of intra-islet paracrine pathways also signal through GPCRs (Winzell and Ahrén, 2007; Ahrén, 2009), some of which critically depend on the primary cilium. These include receptors for locally secreted neurotransmitters and endocrine hormones. β-cells produce in addition to insulin several neurotransmitters including serotonin (5-HT) and γ-aminobutyric acid (GABA) (Almaça et al., 2016; Arrojo E Drigo et al., 2019; Huising, 2020; Parveen et al., 2022). 5-HT is packaged in insulin vesicles for co-release with insulin and promotes glucose clearance in the islet *via* stimulation of insulin and inhibition of glucagon release (Bennet et al., 2015; Almaça et al., 2016). The 5-HT receptor 5HT_6_ (HTR6) is a ciliary GPCR that is expressed in islets and has a well-characterized ciliary localization sequence that has made it a commonly used ciliary-targeting marker (Barbeito et al., 2021; Barbeito and Garcia-Gonzalo, 2021). GABA is another β-cell-derived neurotransmitter, a protein product of the enzyme GAD that is also an autoantigen in type 1 diabetes. GABA serves to inhibit glucagon secretion of α-cells under hyperglycemic conditions (Rorsman et al., 1989), and a recent report demonstrates that GABA signaling is mediated by the ciliary GABA-B1 receptor where it coordinates local Ca^2+^ signaling (Sanchez et al., 2022). Islet α-cells secrete glucagon along with the neurotransmitter acetylcholine (ACh), both of which serve to amplify insulin secretion from β-cells and potentiate β-cell responsiveness to repeated glucose stimulation (Samols et al., 1965; Satin and Kinard, 1998; Rodriguez-Diaz et al., 2011; Capozzi et al., 2019; Noguchi and Huising, 2019; Huising, 2020). While the glucagon and muscarinic ACh receptors, both GPCRs, have not been described in cilia, it remains a curious question whether these paracrine receptors could also be functionally if not physically associated with cilia in islet cells. Finally, δ-cells secrete somatostatin, a local inhibitor of glucagon and insulin secretion (Taborsky and Ensinck, 1984; Rorsman and Huising, 2018; Noguchi and Huising, 2019; Huising, 2020), whose signaling is mediated by a group of somatostatin receptors (SSTRs), GPCRs with specialized expression on islet cells (Kumar et al., 1999; Barbeito and Garcia-Gonzalo, 2021). We and others have shown that SSTR3 is strongly localized to β-cell primary cilia (O’Connor et al., 2013; Cho et al., 2022), where we postulate that it mediates β/δ-paracrine crosstalk *via* somatostatin. While the presence of primary cilia on δ-cells has been reported by microscopy studies (Yamamoto and Kataoka, 1986; Aughsteen, 2001; Zhang et al., 2005; Lodh et al., 2014), little is known about their function. What has been demonstrated convincingly is that δ-cells extend long, secretory filopodia toward other endocrine cells to form dynamic interactions and contacts, an analogous structure to *motile* primary cilia with a proposed role for enhancing the range and efficiency of δ-cell paracrine inhibition (Arrojo E Drigo et al., 2019; Moruzzi et al., 2022).

### Calcium signaling

In the past two decades, the use of genetically encoded calcium biosensors and electrophysiological measurement of localized ion currents have allowed direct interrogation of ciliary versus global calcium dynamics. At this point, we understand primary cilia to be specialized calcium signaling compartments that contain measurable Ca^2+^ dynamics (DeCaen et al., 2013; Delling et al., 2013; Nauli et al., 2016; Pala et al., 2017). In turn, Ca^2+^ as a signaling molecule modulates the activity and dynamics of the cilium itself (DeCaen et al., 2013; Delling et al., 2013; Doerner et al., 2015). The expression of Ca^2+^ channels in the ciliary compartment is likely cell type-specific and dynamically regulated (Raychowdhury et al., 2005; DeCaen et al., 2013; Delling et al., 2013; Nauli et al., 2016). In pancreatic islets, where glucose-stimulated ciliary Ca^2+^ has been shown to be uncoupled from that in the cytosol (Sanchez et al., 2022), the presence and function of primary cilia nevertheless appears required for normal cytosolic Ca^2+^ fluxes (Hughes et al., 2020; Cho et al., 2022). In β-cells, in particular, both glucose-stimulated cytosolic Ca^2+^ and the downstream insulin secretion depend on the cell having an intact, *motile* primary cilium, as evidenced by experimental models of IFT88-mediated cilia deletion and dynein-mediated cilia dysmotility (Hughes et al., 2020; Cho et al., 2022). β-cells are heterogeneous in their cytosolic Ca^2+^ response, with distinct subsets of cells exhibiting higher levels of excitability or functional connectivity (Johnston et al., 2016; Westacott et al., 2017; Salem et al., 2019; Dwulet et al., 2021; Kravets et al., 2022). Targeted ablation or inhibition of these functional nodes can perturb whole-islet Ca^2+^ oscillations, suggesting that there may be cells capable of driving neighboring cell Ca^2+^ dynamics and thereby coordinating pulsatile insulin secretion (Johnston et al., 2016; Westacott et al., 2017; Salem et al., 2019). The potential role of primary cilia underlying the heterogeneous Ca^2+^ behavior in β-cell populations remains unexamined and represents an area that could add further granularity to our understanding of islet physiology. Given the recent observation that β-cell cilia motility is tied into the cytosolic Ca^2+^ response (Cho et al., 2022), the examination of cilia dynamics in functionally distinct β-cell subsets may be particularly relevant and may reveal new mechanisms by which cilia regulate β-cell excitability, calcium handling, and connectivity.

### Cyclic AMP

Cyclic AMP (cAMP) is another important soluble messenger with compartmentalized ciliary production and function distinct from those in the cell body. In a variety of cells, primary cilia act as a cAMP signaling micro-domain by sequestering GPCRs that generate and signal through cAMP in a local regulatory circuit, tuning cellular responses to hormones, neurotransmitters, and other factors. Spatial control of ciliary cAMP signaling is achieved at multiple levels, including cAMP generation by cilia-localized adenylyl cyclases (AC) 3, 5, and 6, cAMP signal transduction through effector enzymes EPAC and PKA, signaling mediators β-arrestin, and negative regulators such as Gpr161 and ciliary phosphodiesterases (Kovas et al., 2008; Choi et al., 2011; Mick et al., 2015; Bachmann et al., 2016; Green et al., 2016). Some of these components of the cAMP signaling pathway are stable cilium-resident proteins in islets, such as AC3, while others only become detectable when ciliary exit is impaired, suggesting that these proteins may be present in the cilium in low copy numbers or have a high ciliary turnover rate (Mick et al., 2015; Wu et al., 2021). Among ciliary cAMP signaling pathways, the best studied is Hedgehog (Hh) signaling, where the consensus view is that the cilium maintains a resting cAMP level in absence of Hh ligand, leading to PKA-dependent phosphorylation of Gli transcription activators and repression of Hh signaling until ligand engagement (Moore et al., 2016; Jiang et al., 2019; Hansen et al., 2022). Cilia regulate endogenous Hh activity, which is important in pancreatic cell proliferation and best studied in the exocrine pancreas, particularly in the setting of ductal cell tumorigenesis. In both the developing pancreas and mature pancreatic epithelium, cilia-localized Gli2 regulates Hh signaling, whereas elimination of cilia and ectopic expression of non-suppressor Gli2 lead to undifferentiated exocrine tumors and reduced functional exocrine and endocrine tissue mass (Pasca di Magliano et al., 2006; Cervantes et al., 2010). Of note, while the ciliary dependence of the Hh pathway is well-established, the observation of a higher ciliary basal [cAMP] over cytosolic [cAMP] is not universal and may be dependent on cell type and experimental system (Moore et al., 2016; Jiang et al., 2019).

In pancreatic islets, despite the hundreds of β-cell GPCRs that have been identified in human transcriptome and proteome studies, less than a handful have confirmed ciliary localization, and among these, few have been shown to generate local ciliary cAMP gradients (Wu et al., 2021). Two such receptors are FFAR4 and PTGER4, which respond to their cognate agonists omega 3 free fatty acid and prostaglandin PGE_2_ to produce ciliary cAMP, presumably *via* localized pools of AC3. This then signals through the cAMP effectors EPAC and PKA to modulate downstream insulin release (Wu et al., 2021). Other well-known β-cell G-coupled receptor agonists include glucagon and glucagon-like peptide 1 (GLP-1), both of which serve to amplify insulin secretion triggered by glucose (Furman et al., 2010; Tengholm, 2012; Stožer et al., 2021; Senatore et al., 2022), but their receptor location has not been implicated in cilia. Studies of ciliary GPCR signaling in islet cells may be sensitive to detection methods and face other technical hurdles including limited biomaterial availability of islet cilia (one per cell), balancing sensitivity vs. specificity in imaging sensors, stacked against the low abundance or transient localization of the signaling proteins themselves. Thus, as in the investigation of ciliary Ca^2+^ dynamics, there likely remain additional ciliary GPCRs important for cAMP regulation that await discovery in islet cells (Table 1). Characterization of ciliary vs. cytoplasmic cAMP dynamics, their relative contribution to cellular signaling, mechanisms of ciliary restriction of cAMP signaling components, and identification of additional islet cell-specific ciliary GPCRs and their signaling characteristics will aid our understanding of glucoregulatory pathways and inform drug discovery in diabetes.

**TABLE 1:** Endogenous signaling proteins known to localize to islet cell cilia. A survey of recent literature reveals multiple classes of receptors and signaling molecules whose ciliary expression as *native* proteins has been identified in primary islets or cell lines, and whose function go beyond maintaining ciliary structure e.g. IFT88, tubulin. Also not listed are proteins whose ciliary localization have been demonstrated *via* tagged constructs e.g. 5HT6, KISS1R, and Gli2 (Cervantes et al., 2010; Wu et al., 2021; Sanchez et al., 2022), while their endogenous ciliary expression have not yet been reported. Many more cilia signaling proteins and pathways have been described in extra-pancreatic cell types and likely await discovery in islets.

Ciliary protein	Function	First description in islet cells
IR (insulin receptor)	Insulin autocrine signaling	Gerdes et al. (2014)
SSTR3 (somatostatin receptor 3)	GPCR, putative paracrine signaling	Iwanaga et al. (2011)
FFAR4 (free fatty acid receptor 4)	GPCR, fatty acid signaling	Wu et al. (2021)
PTGER4/EP4 (prostaglandin E receptor 4)	PGE2, Gα_s_ and cAMP production	Wu et al. (2021)
LKB1 (liver kinase B1)	β-cell polarity, tumor suppressor	Boehlke et al. (2010)
AC3 (adenylyl cyclase 3)	cAMP generation	Wu et al. (2021)
ARL13B (ADP-ribosylation factor-like protein 13B)	GTPase, hedgehog signaling	Gerdes et al. (2014)
Smo (Smoothened)	Hedgehog signaling	Sanchez et al. (2022)
Ptch (Patched)	Hedgehog signaling	Sanchez et al. (2022)
GABA_B1_ (γ-aminobutyric acid receptor B1)	GABA signaling, ciliary Ca^2+^	Sanchez et al. (2022)

### Cilia, vasculature, and hypoxia

The islet vasculature richly interacts with endocrine cells (Bonner-Weir et al., 2015; Dybala et al., 2020; Dybala and Hara, 2021), where primary cilia play an increasingly recognized role in between. Primary cilia sense both mechanical and chemical signals, responding to vascular flow and content and transmitting these signals into the cell (Pala et al., 2018; Ma and Zhou, 2020). Endothelial cells depend on ciliary signaling pathways such as Wnt, Hedgehog, and Notch, to maintain the vascular barrier in the heart, kidney, and the blood-brain barrier (Patschan et al., 2016; Pala et al., 2018; Ma and Zhou, 2020; Mentor and Fisher, 2022). In islets, primary cilia help establish and maintain the microvasculature which is critical to nutrient and oxygen delivery to β-cells (Gerdes et al., 2014; Xiong et al., 2020). In particular, β-cells secrete VEGF-A during islet development and during hypoxia to promote the formation of fenestrated capillaries (Vasir et al., 1998; Vasir et al., 2000; Lammert et al., 2003; Brissova et al., 2006; Reinert et al., 2013), a process now known to be partly mediated by ciliary VEGF receptors on endothelial cells (Wang et al., 2017; Xiong et al., 2020). The absence of functional cilia in Bbs4 and Pifo knockout mouse models delays islet revascularization during transplant engraftment and alters vessel density, diameter, and permeability, impairing the islet’s ability to fine-tune glucose metabolism (Gerdes et al., 2014; Xiong et al., 2020). Islets lacking β-cell cilia in the βCKO mouse have reduced islet cell mass and particularly β-cell mass (Hughes et al., 2020), which may result from either decreased proliferation or accelerated β-cell loss or both. The related β-cell cilia knockout mouse βICKO showed no ill effects in vascular formation but nonetheless produced similar metabolic impairments including reduced insulin secretion and glucose tolerance (Volta et al., 2019; Xiong et al., 2020). Whether the endocrine defects in these β-cell cilia knockout models are related to disrupted blood vessel function, if not their formation, remains to be seen.

The primary cilium has multiple roles in islet hypoxia regulation related to its effects on islet size determination, islet cell survival, and metabolic adaptation (Komatsu et al., 2017; Muthyala et al., 2017; Sankar et al., 2019; Catrina and Zheng, 2021). The major hypoxia regulator protein von Hippel-Lindau (VHL) is localized to the ciliary axoneme where it controls microtubule dynamics, cilia stability, and downstream actions of glycogen synthase kinase (GSK) 3β, Aurora kinase A (AURKA), and hypoxia-inducible factor (HIF) (Thoma et al., 2007; Lolkema et al., 2008; Yang et al., 2019; Chowdhury et al., 2021). These downstream signaling pathways also work in concert to maintain cilia functional integrity, the disruption of which affects cell cycle control. Thus, in addition to regulating angiogenesis and vascular dilatation, primary cilia may control hypoxia responses that affect islet cell decisions and properties, while the ciliary localization of key hypoxia regulatory proteins await confirmation in islets. Overall, the emerging evidence is suggestive of primary cilia regulating cell size and density, nutrient sensing and metabolism, electrical synchrony, and endocrine-exocrine communication, all dynamic processes that depend on a functional crosstalk between islets cells and vasculature and are continually remodeled in the adult pancreas (Meda et al., 1984; Atwater and Sherman, 1993; Granot et al., 2009b; Roscioni et al., 2016; Dybala et al., 2020; Cottle et al., 2021). In rodent and human islets, groups of β-cells are in intimate contact with endothelial cells (Weir and Bonner-Weir, 1990; Granot et al., 2009b; Hogan and Hull, 2017). At least in rodents, β-cell primary cilia appear to be polarized away from the vascular apogee (Gan et al., 2017), suggesting that the route of communication may not be direct physical contact between β-cell cilia and endothelial cells. A number of secreted factors could play a role in islet endocrine-endothelial cell crosstalk, particularly endothelin-1 (Hogan and Hull, 2017), and clarifying whether this signaling loop occurs *via* primary cilia would help further define the reciprocal regulation between these islet cell populations.

## Concluding remarks

Primary cilia are a diminutive but vital cell-surface structure that promotes cellular function and interaction with the microenvironment. Recent studies reveal new and surprising roles for pancreatic islet cilia in glucose homeostasis involving both endocrine and non-endocrine cells. While we and others have begun to elucidate the mechanisms of cilia-dependent islet cell secretion and paracrine communication, many other areas await further study, including islet cilia GPCR signaling, the role of second messengers, cilia regulation of β-cell polarity, size, and energy metabolism, as well as bidirectional communication among endocrine and non-endocrine cells *via* their cilia. Future studies exploring these new areas may reveal ciliary targets to treat human diabetes and metabolic diseases.
